# Opportunities and challenges of pain-related myocardial ischemia-reperfusion injury

**DOI:** 10.3389/fphys.2022.900664

**Published:** 2022-09-02

**Authors:** Wenhua Jiang, Yue Yin, Xiaoming Gu, Zihui Zhang, Heng Ma

**Affiliations:** ^1^ Institute of Medical Research, Northwestern Polytechnical University, Xi’an, China; ^2^ Department of Physiology and Pathophysiology, Fourth Military Medical University, Xi’an, China

**Keywords:** pain, myocardium, cardiovascular diseases, myocardial ischemia reperfusion, cardioprotection

## Abstract

Pain is one of the most serious problems plaguing human health today. Pain is not an independent pathophysiological condition and is associated with a high impact on elevated disability and organ dysfunction. Several lines of evidence suggested the associations of pain with cardiovascular diseases, especially myocardial ischemia-reperfusion (I/R) injury, while the role of pain in I/R injury and related mechanisms are not yet comprehensively assessed. In this review, we attempted to explore the role of pain in myocardial I/R injury, and we concluded that acute pain protects myocardial ischemia-reperfusion injury and chronic pain aggravates cardiac ischemia-reperfusion injury. In addition, the construction of different pain models and animal models commonly used to study the role of pain in myocardial I/R injury were discussed in detail, and the potential mechanism of pain-related myocardial I/R injury was summarized. Finally, the future research direction was prospected. That is, the remote regulation of pain to cardiac function requires peripheral pain signals to be transmitted from the peripheral to the cardiac autonomic nervous system, which then affects autonomic innervation during cardiac ischemia-reperfusion injury and finally affects the cardiac function.

## 1 Introduction

The International Association for the Study of Pain (IASP) redefined pain as “an unpleasant sensation or emotional experience associated with actual or potential tissue damage, or described in terms of such damage” in 2020. This definition, which downplays the description of tissue damage in favor of individual reports, is universally applicable to all pathophysiological pain manifestations (Collaborators, 2016). Pain can be broadly divided into nociceptive, inflammatory, and pathological pain, either spontaneous or induced (Woolf, 2010). According to the duration of pain, pain can be divided into transient, acute, or chronic pain as well. Transient pain refers to programmed pain caused by the activation of pain sensors when the body is injured by excessive external stimuli, which has protective effects and medical intervention is not required. Acute pain focuses on the activation of pain transmitters after the body has suffered local damage, and heals spontaneously without specific therapy. Patients are diagnosed with chronic primary pain when pain lasts for more than 3 months accompanied by significant emotional abnormalities or dysfunction (Loeser and Melzack, 1999). In the International Classification of Diseases revised by the World Health Organization in 2018, chronic pain was listed as an independent disease for the first time. Since then, pain as a disease has attracted extensive attention and research around the world (Barke et al., 2021).

Pain is an extremely common health problem in modern society. About 17,000 people born in England, Scotland and Wales took part in a pain study at the age of 45. The results showed the overall prevalence of pain has reached 53%, including 10% with chronic neuralgia (Vandenkerkhof et al., 2011). Bouhassira et al. showed that the prevalence of moderate to severe chronic pain in the general population was 19.9% (Bouhassira et al., 2008). Patients with chronic pain have a high mortality rate. Chronic pain is one of the major global disease burdens (Torrance et al., 2006; Bouhassira et al., 2008; Phillips, 2009; Macfarlane et al., 2017). Chronic pain leads to central and peripheral pathophysiological changes and is regulated by multiple social, psychological, and physiological factors (Dominick et al., 2012). More importantly, long-term chronic pain gives patients very poor emotional experience and psychological burden. However, analgesics for pain symptoms such as non-steroidal drugs have obvious cardiovascular toxicity (Breivik et al., 2006; Colombo et al., 2006; Scholz et al., 2019). Individuals suffering from pain often follow an unhealthy diet and lifestyle that increase the risk of death from factors such as low levels of physical activity, malnutrition, sleep problems, and substance abuse, all confer an increased the risk of cancer and cardiovascular disease (Andersson, 2009; Vandenkerkhof et al., 2011).

Cardiovascular diseases (CVD), including stroke, congenital heart disease, arrhythmias, coronary heart disease, heart failure, valvular disease, venous disease, etc., rema in the leading cause of death worldwide causing enormous global health and economic burden (Heusch and Gersh, 2017; Reed et al., 2017; Benjamin et al., 2019; Mensah et al., 2019). The prevalence and mortality of CVD continue to rise worldwide except in developed countries (Roth et al., 2020). CVD is influenced by environmental and genetic factors. Hypertension, obesity, diabetes, high blood cholesterol levels, smoking, alcohol consumption, lacking exercise all increase the risk of CVD (Mathers and Loncar, 2006; Forget, 2016; Kyu et al., 2016; Timmis et al., 2018). Emerging research suggests that pain is also strongly linked to CVD.

Accumulating evidence showed that pain and CVD share a common genetic basis (Winsvold et al., 2015a; Pickrell et al., 2016; Winsvold et al., 2017a; van Hecke et al., 2017). The large-scale genome-wide association study (GWAS) of CAD (coronary artery disease) and migraine pointed out the common susceptibility gene locus PHACTR1 (encoding phosphatase and actin regulator 1 protein) of the common mechanism of the two diseases (Winsvold et al., 2017a). Chronic pain and CVD often occur simultaneously, but the mechanisms of comorbidities remain unclear (Birnbaum et al., 1997; Winsvold et al., 2015b; Winsvold et al., 2017b; Tesarz et al., 2019; Booker and Content, 2020). The relationship between pain and CVD is not well defined. Acute pain has protective effects on myocardial ischemia-reperfusion (I/R) injury (Basalay et al., 2012; Redington et al., 2012; Redington et al., 2013; Cheng et al., 2017). However, long-term chronic pain causes the myocardium to be more vulnerable to I/R injury (Li et al., 2018; Yang et al., 2018; Tesarz et al., 2019). The purpose of this review is to provide a better comprehension of the association between pain and myocardial I/R injury. We first introduce the existing pain models and the limitations of animal models, and then summarize that acute pain may be a protective way of myocardial ischemia-reperfusion injury, while chronic pain may aggravate myocardial ischemia-reperfusion injury. Finally, we also illustrate the limitations of clinical research and future research directions: changes of autonomic nervous system in pain related myocardial ischemia-reperfusion injury.

## 2 Animal models for pain-related cardiovascular disease studies

Pain is defined as a disease that seriously affects human health. At present, few studies on pain treatment have been translated into clinical treatment. We all know the lack of translational progress in the field of pain (Mogil, 2009; Abboud et al., 2021). The understanding of pain is still evolving. The construction of pain models in animals makes it more convenient for pain-related research. Table 1 summarizes common animal pain models in studies, as well as the modeling process and respective characteristics.

**TABLE 1:** A summary of the different pain models and their characteristics.

Study	Species	Model	Protocol	Main features
**Neuropathic pain**				
(Bennett and Xie, 1988)	rat	chronic constriction injury (CCI) of the sciatic nerve	• anesthetize	• post-operatively, the rats show hyperalgesia, ectopic pain, and spontaneous pain
			• expose the sciatic nerve	• the pain lasts more than 2 months
			• use 4–0 catgut to encircle the sciatic nerve and make 4 mild binding rings	
			• suture surgical incision	
			• pain test	
(DeLeo et al., 1994)	rat	sciatic nerve freezing injury model	• anesthetize	• autophagy and pain were abnormal after surgery
			• expose the sciatic nerve	• abnormal pain lasts for about 3 weeks
			• the cryoprobe performs a freeze-thaw-freeze cycle on the sciatic nerve	• the damage is reversible
			• behavioral observation	
(Decosterd and Woolf, 2000)	rat	nerve injury model with sciatic nerve branches preserved	• anaesthetize	• hyperalgesia to mechanical and thermal stimuli is observed postoperatively
			• expose the sciatic nerve and its three branches	• pain lasts for more than 7 weeks
			• the sural nerve is intact, and the common peroneal nerve and the tibial nerve are ligated with 5–0 filament	
			• behavioral test	
(Ho Kim and Mo Chung, 1992)	rat	spinal nerve ligation (SNL)	• anesthetize	• mechanical pain persistes for 10 weeks after the operation
			• one side of the paravertebral muscle is excised at the level of L4 ∼ S1	• no autophagy
			• excise the L6 transverse process, and separate the spinal nerves from L4 to L6	• the operation procedure is fixed and the model is relatively stable
			• ligation the L5 and L6 spinal nerves with a 3–0 thread	
(Hu and Xing, 1998)	rat	Chronic Compression of the Dorsal root ganglion, CCD	• anesthetize	• hyperalgesia with mechanical and thermal stimulation is observed postoperatively
			• expose the L5 intervertebral foramen	• no autophagy
			• a steel rod with a length of 4 mm and a diameter of 0.6 mm is inserted into the L5 foramen of rats to achieve stable compression of the dorsal root ganglion	• hyperalgesia lasts more than 6 weeks
			• suture the muscle and skin	• a model of direct compression of the dorsal root ganglion, which is relatively stable
**Inflammatory pain**				
(Chacur et al., 2001)	rat	sciatic inflammatory neuritis (SIN)	• anaesthetize	• Mechanical hyperalgesia developed after the operation, but no hyperalgesia to heat
			• the sciatic nerve is exposed and zymosan is injected around the sciatic nerve	
(Hunskaar and Hole, 1987)	albino mice	formalin induced pain model	• 1% formalin is injected subcutaneously into the dorsal part of the mice hind foot	• the pain behavioral response is the time it takes the mouse to lick the injected paw
				• in the early stage (0–5 min) of formalin injection, pain receptors are directly affected, while prostaglandin induced inflammatory pain in the late stage (20–30 min)
(Malcangio and Bowery, 1996)	rat	freund’s adjuvant induced pain model	• 0.5 ml Complete Freund’s adjuvant (CFA) is injected into the articular cavity of the rat hind limbs	• 4 hours after CFA injection, the rats developed local inflammatory response and hyperalgesia to heat stimulation
				• pain behavior can last for 4 weeks
**Pathological pain**				
(Courteix et al., 1993)	rat	diabetes-induced pain model	• intraperitoneal injection streptozotocin	• postoperatively, the rats develope chronic hyperalgesia of heat and mechanical
			• pain test	
Schwei et al. (1999) (Schwei et al., 1999)	C3H/HeJ mouse	pain model of femoral bone cancer	• anesthetizeIncise left knee joint	• mice injected with cancer cells show bone destruction
			• tumor cells NCTC2472 are injected into the femoral cavity	• the animals show harmful behavioral reactions, which are positively correlated with the degree of bone destruction
			• mechanically stimulate experimental animals at 21 days after injection	• the mouse model is able to mimic the pain caused by bone cancer in humans

Pain models mainly include neuropathic pain, inflammatory pain, and pathological pain models (Mogil, 2009). At present, animal models to study the effect of pain on myocardial ischemia-reperfusion injury are only carried out in neuralgia models, such as chronic compression of the dorsal root ganglion (CCD) and spared nerve injury model (SNI). Cheng et al. (Cheng et al., 2017) found that chronic neuropathic pain has a cardioprotective effect in the SNI mouse pain model, which is induced by parasympathetic pathway dependent on the paraventricular thalamus (PVA). Li et al. (Li et al., 2018) established a CCD pain model and found that chronic pain induced aldehyde overload, which in turn caused persistent hyperalgesia and increased MI/R injury. In the study of Yang et al. (Yang et al., 2018), chronic pain was induced by the nerve injury model (SNI). The study found that Melatonin improves TNF- α and inhibits RIP3-MLKL/CaMKII signal-induced necrosis to improve myocardial ischemia vulnerability caused by chronic pain. In the future, the research on pain and cardiovascular disease can be verified in more comprehensive pain models, such as inflammatory pain and pathological pain models.

However, there are still many problems related to the clinical translatability of the animal models. First of all, the behavioral performance of animal models and clinical patient symptoms are significantly different. Translating findings from animal models into clinical studies remains a major challenge (Burma et al., 2017). Secondly, because the experimenters evaluated pain indirectly, the results were highly subjective and varied greatly (Bouali-Benazzouz et al., 2021). The current pain model is inconvenient to observe and evaluate spontaneous pain and persistent pain, but clinical patients are also troubled by persistent pain and spontaneous pain in addition to induced pain. In addition, Clinical pain patients are often accompanied by anxiety, depression, insomnia, and other problems (Zhuo, 2016). When studying the influence of pain on the heart, the influence of anxiety, depression, and insomnia accompanied by pain on the heart function has not been paid attention to. However, there are various clinical causes of pain. For example, some primary diseases or chemotherapy drugs can also cause nerve damage. Therefore, future studies should consider the pathological status of clinical pain and carry out relevant studies in more pain models and consider the effects of accompanying anxiety on cardiac function. In the current studies, they did not explore whether the effect of pain on cardiovascular disease was caused by pain-induced anxiety, so we expect future studies to rule out the effect of anxiety on cardiac function.

## 3 Pain is a protective approach to cardiac ischemia-reperfusion injury that is different from ischemic preconditioning and ischemic postconditioning (Figure 1)

**FIGURE 1:**
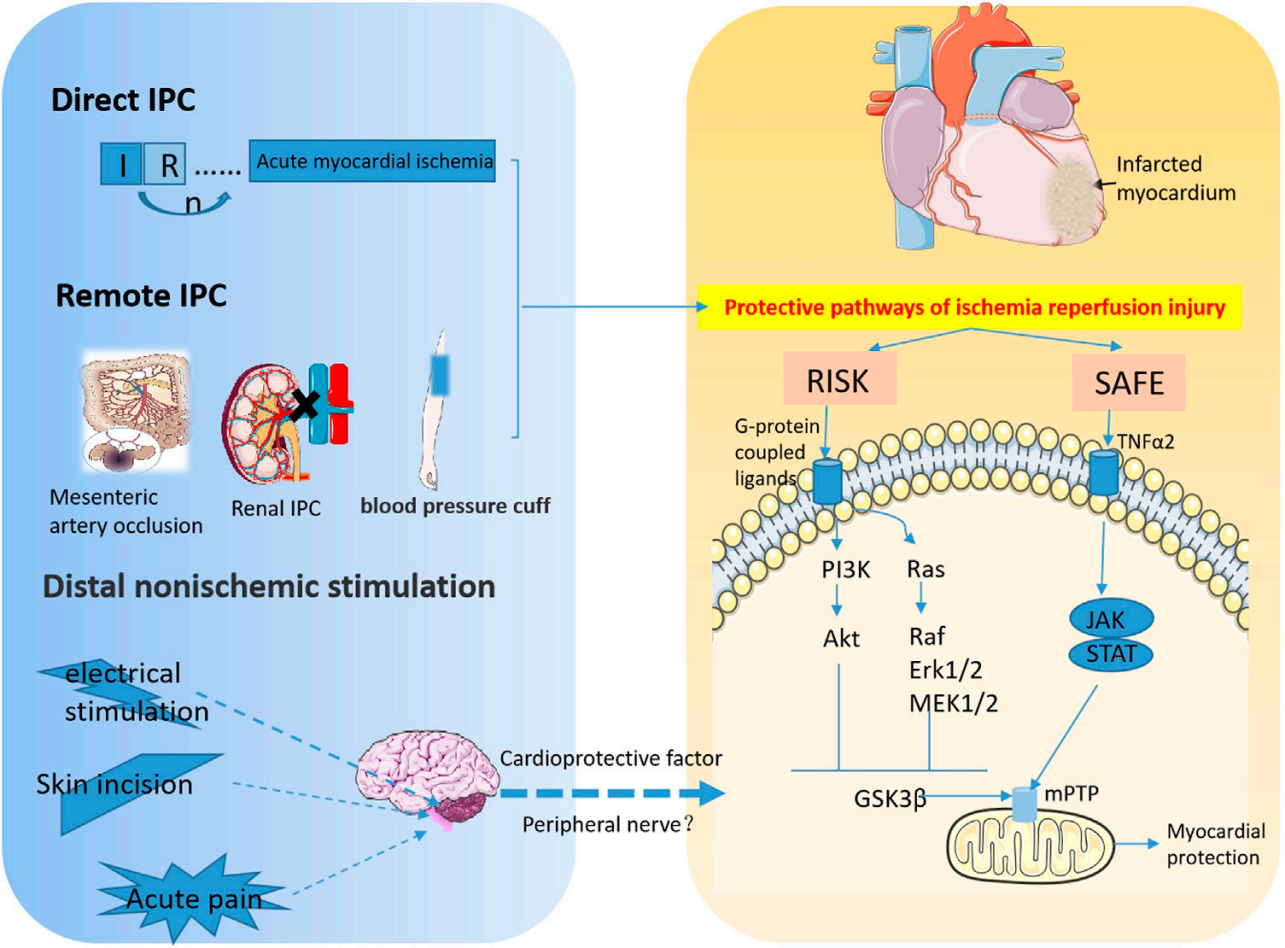
The signaling pathways involved in myocardial IPC and RPCT. This diagram depicts the protective effect of ischemic and non-ischemic preconditioning on acute myocardial ischemia. Transient I/R prior to ischemia attenuated subsequent sustained I/R injury. Remote IPC refers to the ischemic preconditioning of other organs and tissues, such as kidney, mesenteric artery and limbs that besides the heart, that can also protect the myocardium from I/R injury. IPC is mainly involved in myocardial protection through the Reperfusion Injury Salvage Kinase (RISK) pathway, PI3K-Akt and Mek1/2-Erk1/2 pathways, and Survivor Activator Factor Enhancement (SAFE), TNF and JAK-STAT signaling pathways. Distal nonischemic stimuli, including electrical stimulation, skin incisions, and acute pain, improve myocardial I/R injury by stimulating peripheral nerves to release cardioprotective factors into the bloodstream of the myocardium. (RISK, Reperfusion Injury Salvage Kinase; SAFE, Survivor Activator Factor Enhancement; mPTP, mitochondrial permeability transition pore).

### 3.1 Ischemia preconditioning and ischemia postconditioning

It is well known that cardiomyocytes are terminally differentiated cells and cannot regenerate once dead. Therefore, timely restoration of coronary blood flow after myocardial ischemia can maximize the protective effects. At present, the most effective strategy for myocardial ischemia is surgical treatment to achieve coronary artery reperfusion. However, reperfusion inevitably leads to myocardial injury, that is ischemia-reperfusion (I/R) injury (Braunwald and Kloner, 1985; Sharma et al., 2012; Hausenloy and Yellon, 2013; Hausenloy et al., 2016). I/R injury mainly induces endothelial dysfunction, free radical production, nitric oxide consumption, and cytokine release, which leads to myocardial cell death and infarction, cell apoptosis, and autophagy, all ultimately impair cardiac function (Otani, 2008). It is urgent to seek effective therapies to improve I/R injury (Reed et al., 2017).

At present, the endogenous protective modes of myocardial I/R injury are mainly IPC and IPostC (Figure 1). IPC refers to several episodes of transient coronary artery occlusion and reperfusion before ischemia, which can reduce myocardial injury caused by sustained coronary artery occlusion and reperfusion. This protective effect can slow down the process of cell death, but it cannot prevent the end point of cell death (Rezkalla and Kloner, 2004; Heusch, 2015; Basalay et al., 2020). IPC includes direct and remote IPC (RIPC). Direct IPC comprises a few short ischemic-reperfusion sessions before the heart is subjected to sustained ischemia-reperfusion. RIPC is the temporary ischemic treatment of other organs than those that are about to be damaged to tolerate subsequent ischemia (Basalay et al., 2018). These two strategies can protect myocardial cells from sustained I/R injury. Patel et al. found that 15 min or 3 cycles of 5 min mesenteric artery occlusion induces endogenous opioid transport to the myocardium, providing a cardioprotective effect against acute cardiac ischemic events (Patel et al., 2002). Using an acute myocardial infarction (MI) model in the rabbit, Pell’s group found that renal IPC had a significant protective effect against persistent myocardial ischemia. Mechanically, activation of adenosine receptors and K_ATP_ channels is involved in the myocardial protection process (Pell et al., 1998; Liem et al., 2002). Redington’s team was the first to clinically demonstrate the protective effect of remote IPC. The study included 37 children who were about to undergo surgery to repair congenital heart defects. A total of 17 children received a blood pressure cuff for four 5 min cycles of lower limb noninvasive I/R to induce IPC before surgery. The level of troponin I in the experimental group was significantly lower than that in the control group, suggesting that IPC is cardioprotective (Cheung et al., 2006).

Different from IPC, IPostC is a process of several short I/R in the early stage of continuous coronary artery reperfusion, which can reduce the area of MI caused by myocardial I/R injury (Rezkalla and Kloner, 2004; Staat et al., 2005; Mewton et al., 2013; Barsukevich et al., 2015; Hausenloy et al., 2017; Heusch, 2020). Previous studies demonstrate that IPostC reduces the production of reactive oxygen species and reduces oxidation-mediated myocardial injury (Tullio et al., 2013; Hao et al., 2017). IPostC has been demonstrated to induce similar myocardial protective effects as IPC (Heusch, 2015). Most importantly, IPostC can be applied to clinical surgical revascularization (Zhao et al., 2003). Staat et al. demonstrated the beneficial effect of IPostC during coronary angioplasty in acute MI. In this study, 30 patients who underwent coronary angioplasty for acute MI were included. All patients received coronary artery stent reperfusion. Patients in the IPostC group underwent angioplasty balloon therapy of 1-minute dilation and 1-minute contraction within 1 min reperfusion. The release of creatine kinase was significantly reduced in the IPostC group, indicating that the infarct size decreased in the postconditioning group (Staat et al., 2005). Moreover, clinical trials and meta-analyses of Touboul showed that IPost significantly reduced infarct size in patients with acute ST-segment elevation myocardial infarction (STEMI) (Touboul et al., 2015).

### 3.2 Remote preconditioning of trauma alleviates myocardial I/R injury

At present, in addition to IPostC, RPCT and distal pain stimulation have also been shown to alleviate myocardial I/R injury (Figure 1). The myocardial protective effects of nociceptive stimulation are described in detail later (Przyklenk et al., 1993; Takaoka et al., 1999; Tapuria et al., 2008; Robbins et al., 2013; Heusch, 2015).

A few studies showed that distal nonischemic stimulation possessed protective effects on the progression of myocardial ischemia. Gross et al. (Gross et al., 2011) performed abdominal incision preconditioning in dogs to investigate the protective effect of RPCT on persistent myocardial ischemia. The left anterior descending coronary artery (LAD) was occluded 15 min later for 60 min and re-perfused for 3 h. There was a significant reduction of infarct size in MI that received the surgical incision. Further studies found that HOE140, a bradykinin receptor antagonist, eliminated the beneficial effects of RPCT on myocardial I/R injury (Gross et al., 2011). In 2012, another study by Redington et al. found that direct femoral nerve stimulation and topical capsaicin in the rabbit resulted in the release of cardioprotective substances into the bloodstream to induce remote cardiac protection and reduce the infarct size (Redington et al., 2012).

It has also been demonstrated that electrical stimulation can be used as a method of RIPC to alleviate cardiac I/R injury. Birnbaum’s study found that electrical stimulation of the gastrocnemius muscle combined with the reduced flow of the femoral artery significantly reduced myocardial I/R injury in rabbits (Birnbaum et al., 1997). Infarct size was reduced by 65% for both preconditioning combinations compared with sham. However, preconditioning using electrical stimulation of skeletal muscle alone or reduction of femoral artery flow alone does not provide myocardial protection (Birnbaum et al., 1997). To investigate whether electroacupuncture can induce cardiac protection through body fluids, Redington and his colleagues collected plasma after stimulating the Neiguan Point with electroacupuncture in rabbits. The plasma was infused into the heart of another untreated rabbit on the Langendorff perfusion system. After that, the heart was subjected to ischemia for 30 min and reperfusion for 2 h. The infarct size was significantly reduced and myocardial function was improved. It is concluded that electroacupuncture stimulation promoted the release of dialysable cardioprotective factors into the blood to reduce the infarct size caused by myocardial I/R injury, and improve the function of the heart like other remote conditional stimuli (Redington et al., 2013). Merlocco et al. further showed that electrical stimulation of peripheral nerves in rabbits and healthy human volunteers resulted in the release of cardioprotective factors into blood. Similarly, perfusion of isolated hearts improved cardiac function after ischemia (Merlocco et al., 2014).

### 3.3 Acute pain reverses myocardial injury

Non-ischemic surgical stimuli, such as pain from abdominal incisions, activates neurogenic signaling, which ultimately activates cardiac sympathetic nerves to induce cardioprotective subtype PKCε and inhibits the expression of PKCδ, thereby reducing myocardial ischemia injury (Redington et al., 2012; Robbins et al., 2013). As a sympathetic and parasympathetic system blocker, hexamethonium impairs the protective effect of RPCT on MI, while topical capsaicin inducing peripheral pain exerts the protective effect of RPCT on MI by activating C-sensory fibers in the skin.

This study was the first to demonstrate the protective effect of distal nonischemic surgical stimulation against cardiac I/R injury (Jones et al., 2009; Basalay et al., 2012). In conclusion, peripheral nociceptor stimulation has great clinical potential in the treatment of myocardial I/R injury.

To investigate the protective role of neuropathic pain in the myocardium, Cheng et al. (Cheng et al., 2017) established the SNI neuropathic pain mouse model. Then the Left anterior descending coronary artery (LAD) was ligated to achieve myocardial ischemia, and the infarct size in SNI mice was reduced 24 h after reperfusion. Moreover, the expression of creatine kinase muscle and brain isozymes (CKMB) in the SNI group were significantly lower than those in the control and sham group. These results indicated that the I/R injury in the SNI group was less compared with sham groups. In addition, SNI reduced myocardial I/R injury by activating the paraventricular thalamus (PVA) dependent parasympathetic pathways. Further studies showed that pain is not an essential factor for cardioprotection, but PVA plays an irreplaceable role in cardioprotection elicited by neuropathic pain (Cheng et al., 2017). This study was the first to demonstrate that chronic long-distance nonischemic stimulation can provide myocardial protection against I/R injury.

Many clinical studies demonstrated that patients with angina pectoris before acute MI have a better prognosis (Muller et al., 1990; Nakagawa et al., 1995; Ottani et al., 1995; Heusch, 2001; Mladenovic et al., 2008). Retrospective analyses of thrombolytic patients with angina pectoris before MI have shown that patients with angina had smaller infarcts and better outcomes (Heusch, 2001). Interestingly, it has been shown that angina did not affect the area at risk for acute MI, but reduced and slowed down the process of cell death (Yamagishi et al., 2000). The possible mechanism by which angina pectoris improved the prognosis of patients with MI could be the development of collateral vessels caused by chronic angina pectoris (Pérez-Castellano et al., 1998; Fujita et al., 1999; Yamagishi et al., 2000; Rezkalla and Kloner, 2004). However, angina pectoris within 24 h before infarction acted as a preconditioning role to improve cardiac function (Rezkalla and Kloner, 2004). A clinical study found that angina pectoris before infarction in 78 patients with acute MI improved contractile function. Angina pectoris had protective effects on the myocardium of patients with MI before reperfusion (Iglesias-Garriz et al., 2005).

## 4 Chronic pain aggravates myocardial I/R injury

A number of previous clinical and epidemiological studies reported a higher cardiovascular mortality rate in patients with chronic pain, and pain is a risk factor for cardiovascular disease (McBeth et al., 2009; Torrance et al., 2010; Goodson et al., 2013; Fayaz et al., 2016; Tesarz et al., 2019). Jonas Tesarz et al. analyzed data from the Longitudinal Multi-Generation Framingham Heart Study cohort and found an approximately 16% increase in all-cause mortality among widespread pain patients, mainly due to cardiovascular events. Moreover, the number of pain areas was also associated with cardiovascular mortality (Tesarz et al., 2019). In 2016, the results of Fayaz’s meta-analysis showed a dose-response association between chronic pain and cardiovascular mortality (Fayaz et al., 2016). However, the effects of chronic pain on cardiovascular disease have been little studied.

**FIGURE 2:**
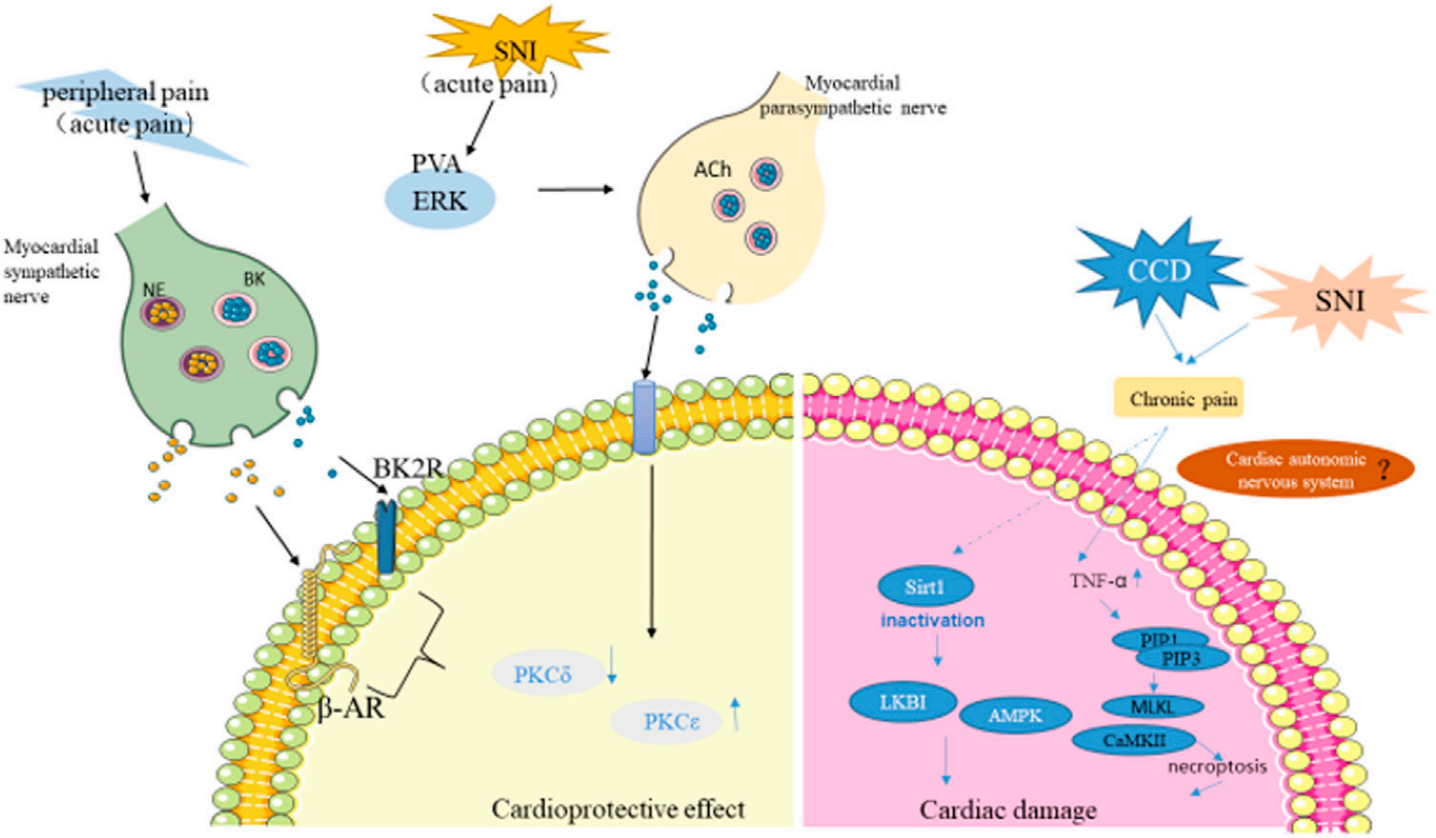
The role of pain in myocardial injury. Acute pain triggered by capsaicin and skin incisions activate C-sensory fibers in the skin, resulting in peripheral pain that activates cardiac sympathetic nerves, further activating PKCε and inhibiting PKCδ to protect the myocardium from I/R injury. Pain activates PVA neurons, which activate cardiac parasympathetic nerves to release Ach(acetylcholine). PKCε is then activated to protect the heart. On the other hand, Chronic pain promotes myocardial ischemia tolerance through SIRT1 carbonyl inactivation and inhibition of LKB1-AMPK interaction. TNF-α overproduction and RIP1-RIP3 interaction were enhanced in chronic pain state, inducing necrosis and further increasing myocardial I/R injury. (NE, norepinephrine; BK, bradykinin; SNI, spared nerve injury model; ERK, the extracellular signal-regulated kinase; Ach, acetylcholine; PVA, the paraventricular thalamus).

To verify the effect of chronic neuralgia on myocardial I/R injury, our group established the mouse chronic compression of the dorsal root ganglion (CCD) model to simulate chronic pain stimulation (Li et al., 2018). In the CCD model, it was confirmed that chronic pain amplifies myocardial I/R injury. The specific mechanism is that chronic pain leads to long-term malignant stress in the body, which inactivated the carbonyl of SIRT1, and eliminated the LKB1-AMPK interaction to promote myocardial ischemia intolerance (Figure 2). In further studies, it was found that myocardium-specific ALDH2 overexpression alleviated chronic pain-induced aldehyde accumulation, thereby alleviating myocardial I/R injury (Li et al., 2018). Later, Yang et al. (Yang et al., 2018) used spared SNI to induce chronic neuropathic pain, and further elaborated that chronic neuropathic pain induced myocardial necrotizing apoptosis by inhibiting the RIP3-MLKL/CaMKII signaling pathway, leading to myocardial ischemia vulnerability. In addition, chronic pain enhanced RIP3-dependent MLKL and CaMKII phosphorylation during myocardial I/R injury. RIP3 knockout inhibited I/R-induced ROS production and myocardial necrosis in SNI mice. Therefore, RIP3-induced myocardial necrosis is a necessary condition for chronic pain resulting in myocardial ischemia vulnerability. Surprisingly, a low dose of melatonin significantly reduced myocardial necrosis in mice with SNI suffering I/R injury, while a high dose of melatonin acted as an analgesic (Yang et al., 2018). These results suggested that chronic nerve pain aggravated myocardial ischemia-reperfusion injury by inhibiting the LKB1-AMPK interaction and the RIP3-MLKL/CaMKII signaling pathway (Figure 1). It provides a possible comorbidities mechanism for exploring chronic pain and myocardial injury, and provides a potential treatment option for intervening myocardial ischemia vulnerability caused by chronic neuralgia pain.

## 5 The effect of pain on myocardial ischemia-reperfusion is related to changes in the cardiac autonomic nervous system

**FIGURE 3:**
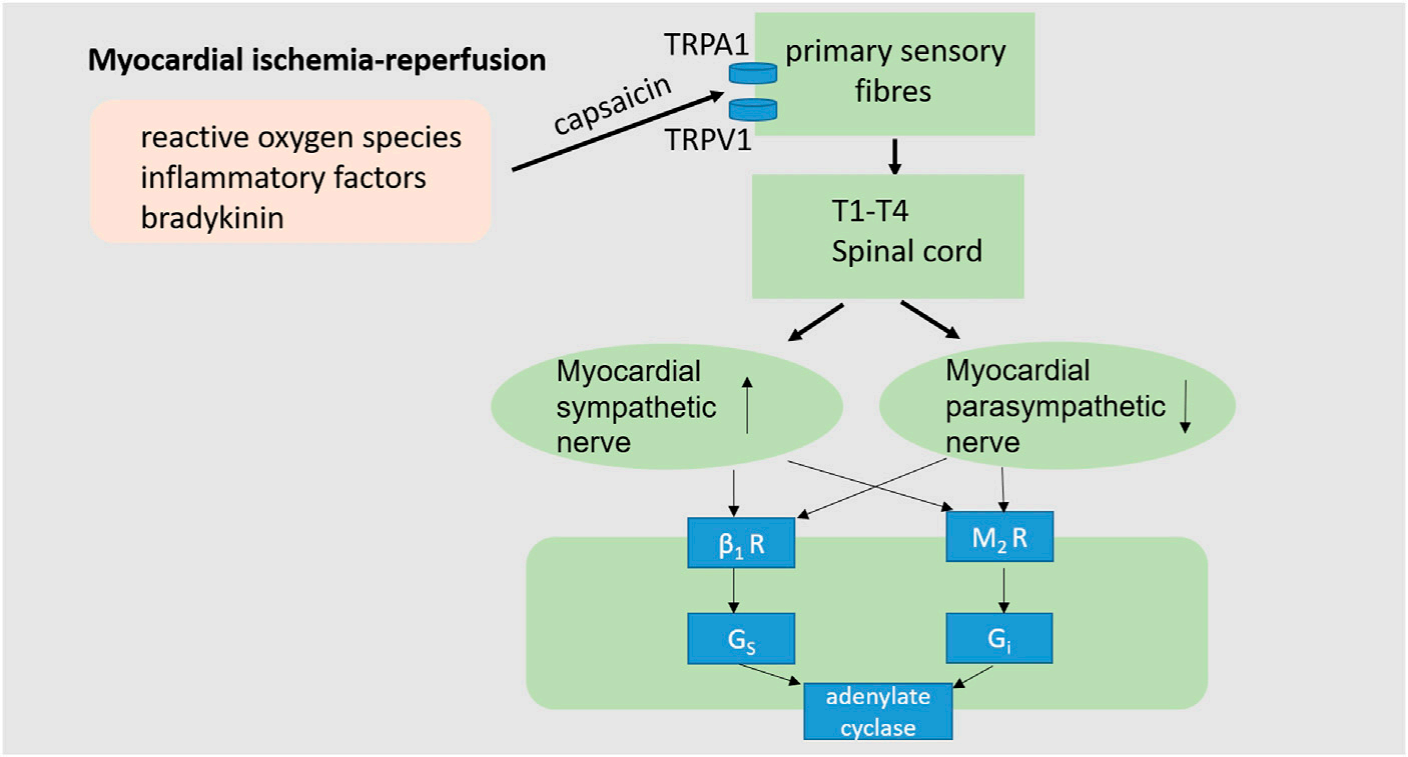
Cardiac neuroregulation associated with myocardial I/R injury. The occurrence of myocardial I/R injury often accompanied by intracellular calcium overload, reactive oxygen species production, inflammatory cytokines and bradykinin, as well as the imbalance of cardiac autonomic nerves, characterized by excessive sympathetic activation and decreased vagal activity, thus impairs cardiac function. (TRPA1, transient receptor potential ankyrin 1; TRPV1, transient receptor potential vanilloid-1; β_1_R, β1adrenoceptor; M_2_R, M2 receptor).

Innervation of the heart plays an important role in regulating cardiac function (Figure 3). It is a complex feedback system that controls the electrical and mechanical functions of the heart. Cardiac innervation consists of intrinsic cardiac ganglion, extracardiac thoracic ganglion, spinal cord, and central nervous region (Ardell and Armour, 2016; Shivkumar and Ardell, 2016). Both intrinsic cardiac ganglion and extracardiac thoracic ganglion include afferent and local circuit neurons and sympathetic post-ganglion efferent nerves, while the intracardial ganglion plexus also contains parasympathetic post-ganglion efferent nerves (Bencsik et al., 2020). The cardiac afferent nerve is connected with the sympathetic nerve and parasympathetic nerve, and can be separated from the autonomic nervous system (Ardell and Armour, 2016; Bencsik et al., 2020).

Normally, the autonomic nervous system is in a state of dynamic balance to maintain healthy cardiac function. During myocardial ischemia and reperfusion, the autonomic nervous system is unbalanced, often manifested as excessive sympathetic stimulation and parasympathetic activity block (Rovere et al., 1998; Thayer and Lane, 2007; Chen et al., 2020). Hypovagal nerve function increases mortality during myocardial ischemia-reperfusion. Myocardial ischemia-reperfusion injury can induce the release of reactive oxygen species, inflammatory factors and bradykinin, which can stimulate the sensory endings of the vagus and sympathetic afferent fibers (Hausenloy, 2003; Thayer and Lane, 2007; Heusch, 2015; Chen et al., 2020). As a non-selective cation channel, TRPA1 (The transient receptor potential ankyrin subtype 1 protein) allows Ca^2+^ and Na^+^ to pass through. TRPA1 plays a role in a range of pathophysiologic processes such as pain, inflammation, and tissue damage and repair (Zygmunt and Hogestatt, 2014; Wang et al., 2019). Activation of TRPA1 on primary sensory neurons generates both afferent and efferent signals. Na^+^ and Ca^2+^ pass through the TRPA1 channel to trigger action potential discharges in cell membranes and neurotransmitter releases in peripheral and central neurites (Zygmunt and Hogestatt, 2014). The central nervous system integrates information from the incoming heart, subsequently increasing cardiac sympathetic activity and suppressing cardiac vagal efferent activity (Chen et al., 2020). Thus, strengthening parasympathetic activity and blocking the sympathetic nerve may be a good strategy to enable the autonomic nervous system to play a beneficial role in myocardial ischemia-reperfusion.

Electrical stimulation of the vagus nerve is known to relieve chronic pain, for example, migraine and cluster headaches (electronic devices to stimulate the vagus are currently used in clinics) (Zeng et al., 2015; López-Álvarez et al., 2019). In addition, the vagus nerve stimulation is known to alleviate myocardial I/R injury (Heusch, 2017). Furthermore, parasympathetic activation is known to mediate the infarct-limiting effect of remote ischaemic preconditioning (Mastitskaya et al., 2012). Abdominal incisions induce cardiac protection against myocardial infarction by stimulating peripheral nociception. Nociception triggers neurogenic signaling through the spinal nerve, which activates the sympathetic nervous system in the heart to protect the heart (Jones et al., 2009). Cheng et al. found that chronic neuropathic pain mediated myocardial protection. Nerve injury activates the afferent nociceptive signal pathway through the spinal cord and activates PVA neurons in the brain. PVA neurons then participate in cardiac protection in a vagus dependent manner (Cheng et al., 2017; Cheng and Chen, 2018). Whether the cardiac autonomic nervous system changes in the state of pain, and the consequent changes of the autonomic nervous system’s impact on cardiac function are the issues we pay attention to. Previous studies have confirmed that within 2 weeks after CCI, sympathetic nerve increased cardiovascular function, and then parasympathetic tension dominated (Cheng and Chen, 2018). However, the changes of autonomic nervous system under longer chronic pain need to be further verified. Previous studies by our research group found that chronic pain can aggravate myocardial ischemia-reperfusion injury (Li et al., 2018). However, how pain affects cardiac function is still unknown. Therefore, we speculated that cardiac autonomic nervous system imbalance in chronic pain can lead to impaired cardiac function, and the specific mechanism needs further exploration.

## 6 The challenges in pain-related myocardial ischemia-reperfusion injury

Studies have shown that chronic pain (including chronic localized pain and chronic widespread pain) is a significant risk factor for cardiovascular disease (Winsvold et al., 2015b; Rönnegård et al., 2022). Our research group previously found that chronic pain can aggravate myocardial ischemia-reperfusion injury, but the comorbid mechanism of chronic pain and cardiovascular disease is still being explored (Li et al., 2018; Yang et al., 2018). However, much evidence have proved that acute pain before myocardial ischemia such as angina pectoris, abdominal incision pain, and other non-ischemic stimuli can alleviate myocardial ischemia-reperfusion injury (Jones et al., 2009; Redington et al., 2012). Acute pain and chronic pain play opposite roles in myocardial ischemia-reperfusion injury. At present, we need to solve the following problems: first, how to distinguish and define acute and chronic pain in animal models, such as the specific modeling time and pain scoring criteria. In addition, how to balance the role of acute and chronic pain in myocardial ischemia-reperfusion injury, acute pain plays a dominant protective role or chronic pain plays a more prominent malignant role on the heart (Table 2).

**TABLE 2:** Opportunities and challenges of pain-related myocardial ischemia-reperfusion injury.

Opportunities	Challenges
➢Diversity of pain models	• Pathological complexity of clinical pain and limitations of animal models
➢Acute pain reduces myocardial ischemia-reperfusion injury	• The contradiction between the sexual dimorphism of pain and the current research focusing on male animal models
➢Chronic pain aggravates myocardial ischemia-reperfusion injury	• The difference of pain in different ages and the lack of research on aging individuals
➢Changes of myocardial function caused by imbalance of cardiac autonomic innervation in pain state	• How to balance the effects of acute pain and chronic pain on myocardial ischemia-reperfusion injury?
	• Lack of pain model in cell experiment

Clinically, the etiology and mechanism of pain are complicated. We need to study the comorbidities of pain and cardiovascular disease through animal models and develop analgesic drugs without cardiovascular toxicity. However, the existing pain models are not enough to represent the complex pathologic state of pain (Burma et al., 2017). Some studies have shown that translating the results of animal models into clinical practice has been difficult. Therefore, it is important to construct multiple animal models that correspond to different clinical manifestations of pain. Establishing different animal pain models can explore the relationship between pain and cardiovascular disease from different perspectives.

In addition, Other studies have shown pain-related cardiovascular disease have been carried out in male pain animal models. In epidemiological surveys, women have a higher prevalence of chronic pain (Mogil, 2012). Therefore, the inclusion of female animals in pain studies needs to be considered carefully. The relationship between pain and age is controversial. Studies have shown that pain is more prominent in elderly patients (Helme and Gibson, 2001; Tsang et al., 2008; Macfarlane, 2016; Geltmeier and Fuchs, 2021). In preclinical trials, the aged rats showed more obvious hyperalgesia to knee osteoarthritis (OA) pain. There was no significant correlation between acute pain and age, but there was age and gender difference in chronic pain (Ro et al., 2020). In contrast, studies have shown that pain sensitivity decreases with age (Harkins et al., 1986; Riley et al., 2014; Tinnirello et al., 2021). Due to the lack of clinical data, the influence of age on pain needs to be further explored. However, we should fully consider the relationship between pain and cardiac function at different ages in the study of pain-related cardiovascular disease. Another important question is: how does pain behave in cell experiments? It is also necessary to develop a universal cellular pain model to study the relationship between pain and myocardial ischemia-reperfusion injury.

## 7 Discussion

At present, substantial clinical investigation reveals that chronic pain is associated with cardiovascular disease, but the underlying mechanism remains unclear. Chronic pain leads to an unhealthy lifestyle that increases risk factors associated with cardiovascular disease. The all-cause mortality of patients with chronic pain is increased due to cardiovascular events. While other studies showed that the development of collateral vessels caused by chronic angina pectoris improved the prognosis of patients with MI. The relationship between pain and cardiovascular disease deserves attention.

The endogenous protective measures of myocardial ischemia-reperfusion injury are ischemic preconditioning and ischemic post-conditioning. Cheng et al. (Cheng et al., 2017) found that acute pain can alleviate myocardial ischemia-reperfusion injury in a different way from ischemic preconditioning and post-conditioning. Pain caused by abdominal incisions activates neurogenic signals that ultimately activate the cardiac sympathetic nerve to reduce myocardial ischemia-reperfusion injury. In addition, myocardial I/R injury is mitigated by activation of paraventricular thalamus (PVA) dependent parasympathetic pathways. In conclusion, the autonomic nervous system plays an irreplaceable role in pain-mediated myocardial protection. The different roles of pain in myocardial I/R injury have been studied. Cheng et al. (Cheng et al., 2017) found that myocardial injury was reduced after 5 days of pain, and pain exerted protective effects on injury heart. However, our previous studies found that chronic rational neuralgia aggravated myocardial I/R injury, further impaired cardiac function. A possible explanation is that the chronic pain model we studied was 3 weeks after the pain, whereas Cheng et al. studied changes in heart function after 5 days of pain. The two studies defined chronic pain differently. Different duration and severity of pain may have different pathophysiological effects on cardiac function. The possible explanation is that activation of the PVA prokaryon-dependent parasympathetic pathway during the early stages of pain reduce myocardial I/R injury, whereas prolonged persistent pain has opposite effects. After prolonged *in vivo* malignant stress, the protective signaling pathways in the myocardium were inhibited and the myocardial I/R injury was aggravated, as the consequence, myocardium further damaged. However, the mechanism of chronic pain leading to myocardial I/R vulnerability remains to be further investigated.

Since the autonomic nervous system mediates pain to protect myocardial ischemia-reperfusion injury, whether the myocardial ischemia vulnerability caused by chronic pain is related to the autonomic nervous system imbalance? Whether pain can affect cardiac function by upsetting the balance of the heart’s autonomic nervous system (sympathetic hyperactivity or reduced vagal nerve activity) is a major question for future research. We speculate that chronic pain leads to cardiac sympathetic overactivation and thus affects cardiac function. The specific mechanism is still being explored. Furthermore, the construction of different pain models closer to pathological state would be particularly important for the further study of pain-related CVD.

## Abbreviations

CCD, chronic compression of the dorsal root ganglion; CVD, cardiovascular diseases; IPC, ischemia preconditioning; IPostC, ischemia postconditioning; I/R, ischemia reperfusion; LAD, descending coronary artery; MI, myocardial infarction; RIPC, remote IPC; RPCT, remote preconditioning of trauma; PVA, paraventricular thalamus.
